# PTSD is associated with accelerated transcriptional aging in World Trade Center responders

**DOI:** 10.1038/s41398-021-01437-0

**Published:** 2021-05-24

**Authors:** Pei-Fen Kuan, Xu Ren, Sean Clouston, Xiaohua Yang, Katherine Jonas, Roman Kotov, Evelyn Bromet, Benjamin J. Luft

**Affiliations:** 1 Department of Applied Mathematics and Statistics, Stony Brook University, Stony Brook, NY USA; 2 Department of Family and Preventive Medicine, Stony Book University, Stony Brook, NY USA; 3 Department of Medicine, Stony Brook University, Stony Brook, NY USA; 4 Department of Psychiatry, Stony Book University, Stony Brook, NY USA

**Keywords:** Genomics, Biomarkers

## Abstract

Posttraumatic stress disorder (PTSD) is associated with shortened lifespan and healthspan, which suggests accelerated aging. Emerging evidence suggests that methylation age may be accelerated in PTSD. It is important to examine whether transcriptional age is also accelerated because transcriptome is highly dynamic, associated with age-related outcomes, and may offer greater insight into the premature aging in PTSD. This study is the first reported investigation of the relationship between transcriptional age and PTSD. Using RNA-Seq data from our previous study on 324 World Trade Center responders (201 never had PTSD, 81 with current PTSD, and 42 with past PTSD), as well as a transcriptional age calculator (RNAAgeCalc) recently developed by our group, we found that responders with current PTSD, compared with responders without a PTSD diagnosis, showed accelerated transcriptional aging (*p* = 0.0077) after adjustment for chronological age and race. We compared our results to the epigenetic aging results computed from several epigenetic clock calculators on matching DNA methylation data. GrimAge methylation age acceleration was also associated with PTSD diagnosis (*p* = 0.0097), and the results remained significant after adjustment for the proportions of immune cell types. PhenoAge, Hannum, and Horvath methylation age acceleration were not reliably related to PTSD. Both epigenetic and transcriptional aging may provide biological insights into the mechanisms underpinning aging in PTSD.

## Introduction

Posttraumatic stress disorder (PTSD) is a debilitating psychiatric condition affecting 7% of the US population^1^. It is associated with reduced lifespan and healthspan, suggesting accelerated aging^2,3^. PTSD is a complex disorder with heterogeneous clinical presentation^4^ and is associated with epigenetic modifications potentially resulting from traumatic exposures^5–8^. Recent research has shown that PTSD may be associated with accelerated epigenetic aging^9–12^. The epigenetic clock-based on DNA methylation (DNAm), the most commonly used tool to characterize biological aging, has been found to be a more effective indicator of age-related morbidity and mortality than chronological age^13^. Several epigenetic calculators have been developed to date, including Horvath’s seminal multi-tissue DNAm age calculator^14^ and Hannum’s whole blood DNAm age calculator^15^. Both calculators used the chronological age of healthy samples as a reference. Newer DNAm age calculators have since emerged, aiming to optimize the prediction of phenotypic age according to clinical attributes associated with mortality and morbidity. These include PhenoAge^16^ and GrimAge^17^, which seek to improve the prediction of age-related outcomes, e.g., time to death and time to diagnosis for cancer, Alzheimer’s disease and cardiovascular disease.

While there is intense interest in understanding epigenetic mechanisms for age acceleration, results in the study of PTSD have been equivocal. For example, Wolf et al.^18^ reported that lifetime PTSD severity was not associated with accelerated aging using Horvath’s DNAm age calculator, but was associated with accelerated DNAm aging when computed with Hannum’s DNAm age calculator in US veterans. However, in a follow-up study using a larger cohort where DNAm aging was computed using only Hannum’s calculator, the latter association was not replicated^9^. Instead, PTSD hyperarousal symptoms were found to be associated with accelerated DNAm aging. In a longitudinal study consisting of two time points (T1 and T2), the authors showed that accelerated rate of DNAm aging across T1 and T2 was associated with combined T1 avoidance and numbing PTSD factor scores using Horvath’s calculator but not Hannum’s calculator. Furthermore, no significant association was found for other subdimensions (including hyperarousal) or current PTSD^19^ when using either calculator. In contrast, Boks et al.^12^ reported that PTSD symptoms were negatively associated with DNAm age acceleration in Dutch military personnel, whereas Zannas et al.^20^ did not find a significant association in African American urban population with low socioeconomic status. Additionally, using Horvath’s calculator, Mehta et al.^11^ did not find a significant association between DNAm age acceleration and PTSD diagnosis or severity. Two recent studies in (1) African American urban population using an expanded sample size^21^ and (2) male veterans^22^ showed significant association between GrimAge age acceleration and PTSD. Specifically, Katrinli et al.^21^ found that GrimAge age acceleration was significantly higher in current and lifetime PTSD compared to control, however the results using other DNAm age predictors were not reported. Yang et al.^22^ found that both GrimAge and Horvath age acceleration were significantly higher in PTSD compared to control, however the results were not significant for Hannum or PhenoAge age acceleration. The authors also reported significant positive correlations between changes in GrimAge age acceleration and changes in PTSD symptom severity in a subset of participants with PTSD at baseline, however the correlation was not significant in a subset that included participants without PTSD at baseline. A meta-analysis of nine cohorts did not yield conclusive results^10^. The irreproducible and inconsistent results indicate the limitations of research on DNAm aging in PTSD, and more work in additional cohorts is therefore needed to validate and replicate the current findings.

Although both DNAm and gene expression have been found to be associated with PTSD and are hallmarks for understanding the aging process and associated diseases, no previous study has investigated transcriptional aging in PTSD. One possible explanation is the lack of availability of transcriptional age predictors. In addition, most existing human transcriptional age predictors were developed based on microarray data and are limited to a small number of tissues^23–27^. RNA-Seq has emerged as the current state-of-the-art platform for transcriptional profiling. The only predictor constructed by using RNA-Seq data is the ensemble LDA predictor; however, this predictor was derived only from fibroblast data^28^. Recognizing the gap in existing transcriptional aging research based on RNA-Seq data, our group has recently developed RNAAgeCalc, a multi-tissue transcriptional age calculator^29^, by using the RNA-Seq data from the Genotype-Tissue Expression (GTEx) Program^30^. Our calculator can perform both the across-tissue and tissue-specific transcriptional age prediction. We have further shown that RNAAgeCalc outperforms prior age-related gene expression signatures in predicting age of normal samples and offers complementary information to DNAm age in association analysis of mutation burden and mortality risk across different types of cancer^29^.

The objectives of the current study were twofold: (1) we performed the first investigation of the relationship between transcriptional aging and PTSD, and (2) we compared the different DNAm age calculators for PTSD and correlated these findings to transcriptional aging. To this end, we used *N* = 324 whole blood RNA-Seq gene expression data profiled by using RNA-Seq and matching DNAm data from our previous studies of World Trade Center (WTC) responders^7,8^. We computed the DNAm and transcriptional age and evaluated the associations between PTSD and these biological age predictions.

## Methods

### Participants, DNA methylation, and RNA-Seq

We used matching whole blood DNAm and RNA-Seq gene expression data from our previous studies^7,8,31^ of participants recruited through the Stony Brook WTC Health Program^32^. The current study was approved by the Stony Brook University IRB and written informed consent was obtained from all participants. Master’s level psychologists were trained to administer the PTSD module of the Structured Clinical Interview for DSM-IV (SCID^33^)^34^. Diagnoses were coded as (a) currently meets criteria for PTSD (current PTSD), (b) met criteria since 9/11/2001 but not currently (past PTSD), and (c) did not meet criteria for PTSD (non-PTSD). The SCID was administered concurrently with the blood draw. A total of 324 male participants were profiled (81 with current PTSD; 42 with past PTSD; 201 who never had PTSD). In addition, participants completed the Posttraumatic Stress Disorder Checklist-Specific Version (PCL-17)^35^, a 17-item self-reported questionnaire assessing the severity of WTC-related DSM-IV PTSD symptoms in the previous month. The PCL covers the major domains of PTSD symptoms, including reexperiencing, avoidance, numbing, and hyperarousal. Because cell type proportions have been implicated in whole blood DNAm and RNA-Seq data analysis, the proportions of CD8 and CD4 T cells, natural killer cells, B cells, and monocytes were previously estimated in these samples based on Houseman et al. procedure^36^ on DNAm data. We also included the cell type proportions estimated from RNA-Seq data using CIBERSORT software^37^. Additional details on DNAm and RNA-Seq data preprocessing were described in Kuan et al.^7,8^.

### Epigenetic age calculator

Epigenetic ages from DNAm data were computed with the online DNAm age calculator (http://dnamage.genetics.ucla.edu/), which implements several DNAm age estimators including Horvath^14^, Hannum et al.^15^, PhenoAge^16^, and GrimAge^17^.

### Transcriptional age calculator

Our group has recently developed RNAAgeCalc, a multi-tissue transcriptional age calculator^29^, by using the RNA-Seq data from the GTEx Program, which does not include any of the present samples^30^. On the basis of our previous results indicating that the transcriptional age derived using the 1497 age-related genes identified in Peters et al.^38^ performed best in blood samples, as compared with other age-related signatures^29^, we computed the transcriptional age by using the RNA-Seq data from these 324 WTC responders. Specifically, we used the option in RNAAgeCalc, which was trained on the 1497 genes of the whole blood GTEx RNA-Seq data to compute the transcriptional age on the WTC data.

### Statistical analysis

The correlations between each biological age (i.e., the different DNAm age predictions and transcriptional age) and chronological age were assessed with Pearson correlation coefficients. Age acceleration was defined as the residual by regressing biological age on chronological age. Positive age acceleration indicated a higher biological age than chronological age. A linear model was fitted by using age acceleration as the dependent variable and PTSD diagnosis (current versus never) as the independent variable, with adjustment for chronological age and race. Sensitivity analysis was conducted to compare the results from a model that was further adjusted for the proportions of CD8 and CD4 T cells, natural killer cells, B cells, and monocytes. Analyses were repeated by substituting PTSD diagnosis with total PTSD symptom severity (total PCL) and each of the four dimensions (reexperiencing, avoidance, numbing, and hyperarousal). All continuous variables were standardized in the linear model fit. Univariate associations between each cell type and age acceleration, as well as the associations between cell type proportions estimated on DNAm (Houseman et al. procedure^36^) versus RNA-Seq data (CIBERSORT software^37^) were assessed with Pearson correlation coefficients.

## Results

### Participant characteristics

The mean age of the participants was 51.78 years (SD = 8.12). All were male, most were Caucasian (87.65%), and no significant differences in age or race were observed between cases and controls, as shown in our previous study^7^.

### Correlations among DNAm, transcriptional, and chronological age

**Fig. 1:**
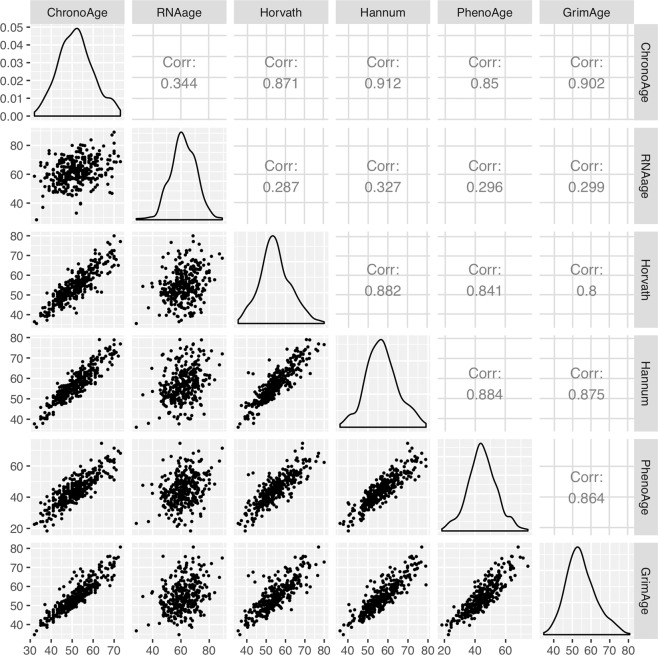
Scatter plots comparing the four DNAm ages (Horvath, Hannum, PhenoAge, and GrimAge), transcriptional age and chronological age. The correlation coefficients between each pair is shown on the upper triangular matrix. All correlation coefficients were significant at *p* < 0.01.

The correlations among the four DNAm age predictions (Horvath, Hannum, PhenoAge, and GrimAge), transcriptional age and chronological age are shown in Fig. 1. In general, the DNAm age predictions highly correlated with chronological age (*r* > 0.8, *p* < 0.01). In contrast, the correlation between transcriptional age and chronological age was lower, at 0.344 (*p* < 0.01). The four DNAm age predictions were also highly correlated among each other (*r* > 0.8, *p* < 0.01). On the other hand, the correlation between transcriptional age and each of the DNAm age prediction was lower (0.28 < *r* < 0.33, *p* < 0.01).

### DNAm and transcriptional age acceleration associated with PTSD diagnosis, PCL, and PCL dimensions

**Fig. 2:**
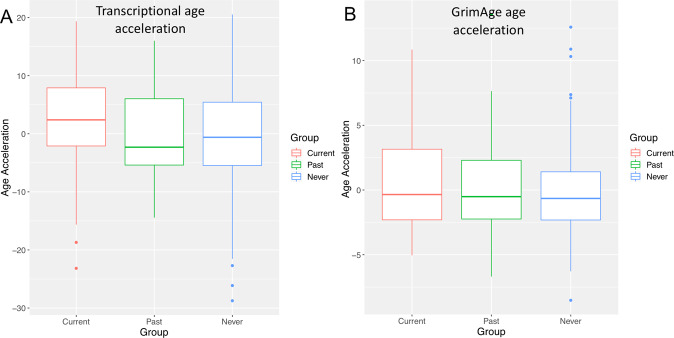
Age acceleration versus PTSD diagnosis. **A** Box plot comparing transcriptional age acceleration to PTSD diagnosis. **B** Box plot comparing GrimAge age acceleration to PTSD diagnosis.

**Fig. 3:**
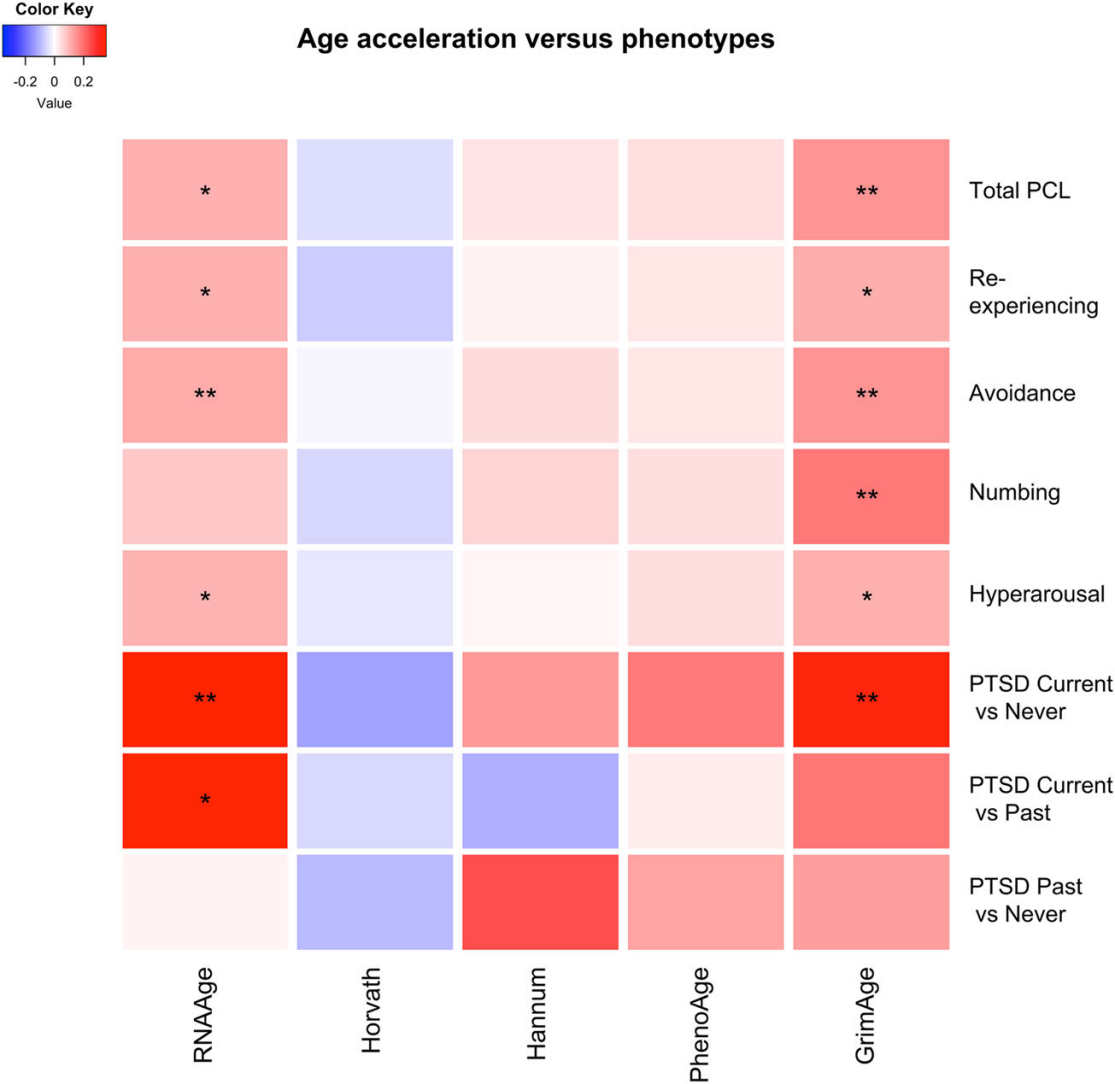
Heat map summarizing the associations between each of the four DNAm age acceleration (column) and PTSD (row) after adjustment for age and race. The color of each cell represents the magnitude of the standardized coefficient β, whereas asterisks indicate *p* value ranges, i.e., **p < 0.05; *0.05 < *p* < 0.1.

The current PTSD group showed higher transcriptional age acceleration than the never PTSD group (*β* = 0.354, *p* = 0.0077) after adjustment for age and race. In contrast, transcriptional age acceleration was not significantly different between current PTSD and past PTSD (*β* = 0.346, *p* = 0.075) as well as between past PTSD and never PTSD (*β* = 0.017, *p* = 0.918), after adjustment for age and race (Fig. 2A). Transcriptional age acceleration was also positively associated with the avoidance dimension (*β* = 0.116, *p* = 0.037) but was not significantly associated with total PCL, reexperiencing, hyperarousal dimensions (0.05 < *p* < 0.1), and numbing (*p* = 0.183) (Fig. 3, Supplementary Table 1).

Among the four DNAm age predictions, only GrimAge age acceleration was significantly higher in the current PTSD group than the never PTSD group (*β* = 0.335, *p* = 0.0097) after adjustment for age and race. GrimAge age acceleration was not significantly different between current PTSD and past PTSD (*β* = 0.190, *p* = 0.386), as well as between past PTSD and never PTSD (0.132, *p* = 0.410), after adjustment for age and race (Fig. 2B). GrimAge age acceleration was also positively associated with total PCL (*β* = 0.146, *p* = 0.010), avoidance (*β* = 0.147, *p* = 0.0083) and numbing dimensions (*β* = 0.186, *p* = 0.012), but was not significantly associated with reexperiencing and hyperarousal dimensions (0.05 < *p* < 0.1) (Fig. 3, Supplementary Table 1).

In contrast, the Horvath, Hannum, and PhenoAge DNAm age acceleration was not significantly associated with PTSD diagnosis, total PCL, or PCL dimensions (Fig. 3, Supplementary Table 1). The results remained consistent after adjustment for cell type proportions (Supplementary Table 1), and transcriptional and GrimAge acceleration remained significantly associated with PTSD diagnosis (*p* = 0.009 and 0.002, respectively). PhenoAge and Hannum methylation age acceleration became associated with current PTSD (*p* = 0.034 and 0.049, respectively), whereas Horvath methylation age acceleration remained not statistically significant after additional adjustment for cell type proportions.

The correlation between estimated cell type proportions from DNAm data (Houseman et al. procedure^36^) versus RNA-Seq data (CIBERSORT software^37^) ranged from 0.583 (monocytes) to 0.707 (CD8 T cells) (Supplementary Table 2). In addition, the associations between transcriptional age acceleration and PTSD diagnosis, total PCL or PCL dimensions were robust with respect to the methods used for estimating cell type proportions as adjustment factors (Supplementary Table 1).

Univariate associations between each cell type and age acceleration showed that DNAm age accelerations were negatively correlated with CD4 T and B cells (*p* < 0.05), whereas the correlations between transcriptional age acceleration and cell type proportions were modest and mostly not significant (Supplementary Table 2).

## Discussion

A range of chronic health conditions are thought to cause accelerated aging including PTSD^39^. Indeed, PTSD has been previously linked in WTC responders to a range of aging-related conditions including memory loss^40^ and increased risk of cognitive impairment^41^ and physical functional limitations^42–44^. There is therefore an effort to develop measures of biological aging for use in this cohort. The current study is the first to examine transcriptional age acceleration in PTSD by using our recently developed transcriptional age calculator, RNAAgeCalc^29^. Our results suggested that WTC responders with current PTSD had evidence of accelerated transcriptional aging compared to WTC responders who did not have PTSD. Interestingly, past PTSD showed no accelerated aging, suggesting that biological age may normalize after this disorder is effectively treated. Notably, we found avoidance was positively associated with transcriptional age acceleration (*p* < 0.05), and further identified consistent trends toward positive associations between total PCL, hyperarousal, reexperiencing dimension, and transcriptional age acceleration, although the strength of the association did not attain statistical significance (*p* < 0.1).

In related work on DNAm aging, Wolf et al.^9^ reported a positive association between DNAm age acceleration and the hyperarousal dimension when using Hannum’s calculator. In their follow-up study on longitudinal samples, however, accelerated rate of DNAm aging across T1 and T2 was only associated with T1 PTSD avoidance and numbing subdimensions when Horvath’s calculator was used, while no significant associations were found in other subdimensions (including hyperarousal) or in PTSD diagnosis^19^. In contrast, our study found that only GrimAge showed significant age acceleration with PTSD diagnosis, total PCL, avoidance and numbing dimensions (*p* < 0.05). Other DNAm age calculators did not show significant statistical associations in the model with adjustment for age and race. After additional adjustment for cell type proportions, all DNAm ages except Horvath’s methylation age were significantly associated with PTSD diagnosis. Additionally, the estimated effect size directions between DNAm age acceleration computed from Hannum and PhenoAge were consistent with the results from transcriptional age and GrimAge; i.e., the estimated standardized coefficients were positive. Unexpectedly, analysis with Horvath’s calculator yielded negative estimated effect size directions in terms of the association between DNAm age acceleration and all PTSD measures considered in this study. A possible explanation for this finding is that Horvath’s method is a multi-tissue calculator trained across multiple non-cancer and cell lines, whereas the other three DNAm age calculators were trained on blood samples. Our study also replicated the findings of the two recent studies which showed that GrimAge age acceleration was significantly associated with PTSD diagnosis^21,22^. GrimAge was constructed based on CpGs associated with smoking and plasma proteins previously found to predict mortality or morbidity, and has been shown to outperform its predecessors in mortality prediction^17^. Taken together, the findings from these three PTSD studies (including the current study)^21,22^ suggest that GrimAge is a powerful epigenetic age predictor in PTSD.

Our results showed that the correlations between transcriptional age and chronological age, as well as between transcriptional age and the four DNAm age were lower compared the correlations between DNAm age and chronological age. This trend was also observed in Peters et al.^38^ and Ren and Kuan^29^, which could be attributed to the different biological mechanisms underlying transcriptional age compared to epigenetic age^38^. For example, age-related genes used in transcriptional age prediction were enriched in RNA metabolism, ribosome biogenesis, purine metabolism, mitochondrial and metabolic pathways^29,38^, whereas the CpGs included in Horvath’s multi-tissue DNAm age calculator were enriched in cell death, cell survival, cellular growth, cellular proliferation, organismal or tissue development and cancer^14^. On the other hand, the CpGs included in PhenoAge were enriched in Kallikreins gene family, methylglyoxal degradation I pathway and polycomb group protein targets^16^. In addition, gene expression exhibits larger biological variability and is regulated by a variety of factors including DNAm^45^, which could contribute to the larger variability observed in transcriptional age prediction.

The negative correlations between DNAm age acceleration and immune cell type proportions (CD4 T and B cells) in this study are in line with results from previous studies which suggest that epigenetic age acceleration is associated with immunosenescence^16,21,46^. On the other hand, immunosenescence (represented by altered estimated cell type proportions in this study) has a weaker association with transcriptional age acceleration.

### Limitations

This study relied on a case–control study of WTC responders with a relatively large sample size with detailed diagnostic information for individuals exposed to a unique event who have been followed-up for nearly two decades. However, central limitations to this work include a lack of specificity about the central systems that may be most affected by transcriptional aging. We focused only on males in this study. Larger samples are needed to investigate the impact of gender differences on transcriptional and DNAm age acceleration in PTSD. Our RNA-seq data was performed on samples derived from whole blood and were thus a mix of cell types. Thus, it remains unclear whether transcriptional and methylation aging are occurring in specific cells or are distributed across all cell types. We controlled for prevalence of cell types statistically, but future work needs to isolate and examine each cell type individually. In addition, while being very well characterized, populations of WTC responders were exposed to a specific set of exposures, thereby potentially limiting generalizability of these results to other populations. Despite the aforementioned limitations this is the first study to identify associations between PTSD and transcriptional age acceleration.

## Conclusion

There is increasing interest in identifying methods for monitoring age acceleration and in identifying reliably correlates of accelerated biological age. This is the first study to establish that transcriptional age acceleration may be associated with chronic PTSD. In addition, results were replicated using DNAm age acceleration computed using the GrimAge algorithm in matched samples. If validated in other cohorts, these findings should advance understanding of the biological mechanisms underlying premature medical morbidity and mortality in PTSD, and aid in identification of potential gene modifiers that may alter the rate of cellular aging in the general population.

## Supplementary information

Supplementary Materials

Supplementary Table 1

Supplementary Table 2

## Data Availability

The RNA-Seq and methylation data were from our previous studies^7,8,31^. RNA-Seq data is available at the Gene Expression Omnibus (accession number GSE97356). Methylation data is available at the Psychiatric Genomics Consortium (PGC) website https://www.med.unc.edu/pgc/.
