# Supplementary material for: PTSD is associated with accelerated transcriptional aging in World Trade Center responders
Source: Transl Psychiatry. 2021 May 24;11:311. doi: 10.1038/s41398-021-01437-0 (PMC8144188; doi:10.1038/s41398-021-01437-0)
Supplement: Supplementary file 1 — Supplementary Materials [file 41398_2021_1437_MOESM1_ESM.docx]

**Supplementary Table 1 Legend.** Associations among DNAm and transcriptional age acceleration and PTSD. Model 1: linear model comparing age acceleration to each clinical variable after adjustment for age and race. Model 2: linear model comparing age acceleration to each clinical variable after adjustment for age, race and cell type proportion estimated from DNA methylation data using Houseman et al. procedure ^1^ . For transcriptional age acceleration, we also included Model 2a: linear model comparing age acceleration to each clinical variable after adjustment for age, race and cell type proportion estimated from RNA-Seq data using CIBERSORT software ^2^.

**Supplementary Table 2 Legend.** Univariate associations between each cell type and age acceleration. For transcriptional age acceleration, we included both the cell type proportion estimated on DNA methylation (Houseman et al. procedure ^1^) and RNA-Seq data (CIBERSORT software ^2^). The associations between estimated cell type proportions from DNA methylation data (Houseman et al. procedure ^1^) versus RNA-Seq data (CIBERSORT software ^2^) were also included.

**References**

1. Houseman EA *et al.* DNA methylation arrays as surrogate measures of cell mixture distribution. *BMC bioinformatics* 2012; **13**(1)**:** 1.

2. Newman AM *et al.* Robust enumeration of cell subsets from tissue expression profiles. *Nat Methods* 2015; **12**(5)**:** 453-457.
